# Acute Glaucoma of Both Eyes Successfully Treated by Paracentesis of the Cornea

**Published:** 1867-11

**Authors:** W. F. Westmoreland

**Affiliations:** Prof. Surgery in the Atlanta Medical College


ATLANTA

Medical and Surgical Journal.

NEW SERIES.

Vol. VIII. NOVEMBER, 1867. No. 9.

### ORIGINAL COMMUNICATIONS.

### ARTICLE I.

*Acute Glaucoma of both Eyes Successfully treated by Paracentesis of the Cornea.* By W. F. Westmoreland, M. D., Prof. Surgery in the Atlanta Medical College.

Since the elaborate memoir by Prof. Von Gräfe of Berlin, in 1857, introducing the operation of the iridectomy for the treatment of Glaucoma, Ophthalmic Surgeons have extensively discussed the pathology of this disease, but as yet have not accurately defined the exact nature of the disease of the eye named "Glaucoma" by iridectomists. Whatever may be its precise character, whether an affection of the Ciliary nerves, a rheumatic condition of the circulation, or a hypersecretion of the fluids of the eye, all, I believe, are disposed to admit that the whole series of phenomena as witnessed in the disease are referable to one cause—"a morbidly increased tension of the tunics of the eyeballs, produced by intraocular (hydrostatic) contained fluids."

It is not, however, my purpose in this article to enter into the discussion of the pathology of this disease, or reproduce the opinions of ophthalmologists who have devoted much time and labor to the investigation of the subject, nor do I propose to discuss the relative merits of the operations paracentesis cornæ, iridectomy and a section of the ciliary muscle, proposed for the relief of this condition of the eye, but simply to report a case which was entirely relieved by tapping the cornea, as suggested and successfully practised by Prof. Sperino of Turin.

In March, 1867, I was called to see Benj. H——, ten years old—well grown and up to the present attack had enjoyed good health. I found him with considerable fever, pain in the head, back and knees, with some cough. I regarded the attack as influenza, which was then prevailing in the city, and prescribed accordingly. Two or three days later I was called again and informed by the mother that he was suffering with his eyes.

Upon examination I found considerable redness of the conjunctiva, but regarded it as a simple catarrhal ophthalmia, and prescribed the ordinary remedies. A week later I was called in great haste and found the family much alarmed. They assured me that the child was entirely blind. Upon examination I found the eyes less congested than when I last saw him, but completely blind in the right eye, and only able to distinguish, imperfectly, large and bright objects with the left eye. I then, for the first time, suspected the true character of the disease. The eyeballs were *tense*; the right perceptibly more so than the left. The pupil of the right eye was greatly dilated, the left not so much so—and slowly responded to a bright light.—The age of the child and its unmanagable disposition precluded the possibility of procuring the ophthalmoscopic and other tests which could be readily obtained in the case of an adult. It was evident that I had a case of acute Glaucoma.

I determined that instead of an immedi ate operation as insisted upon by the majority of ophthalmic surgeons, to try first the power of medication suggested by the surgeons of the Ophthalmic Hospital, Southwark, and put my patient upon large doses of the Iodide of Potassium, and ordinary doses of Belladonna.

The patient, however, continued to grow worse, and at the expiration of ten days was completely blind in both eyes.

It was now evident that an immediate operation was the only hope for my little patient. As Prof. Sperino often performs paracentesis corneæ preliminary to iridectomy, believing that in this way he obviated many of the risks, especially internal hemorrhage, I determined to tap the cornea, and if necessary, a few days later, to perform iridectomy.

On the 8th of April, assisted by Drs. D'Alvigny, Alexander and Flewellen, the patient was placed under the influence of Chloroform, and with a broad-pointed cataract needle, a little curved to prevent wounding the iris, I tapped both eyes, making the incision in the cornea near its junction with the sclerotic. The aqueous humor of both eyes passed out in a jet. The Belladonna and Iodide of Potassium were continued.

No unpleasant symptoms followed, and in four days the patient could very imperfectly distinguish objects, particularly red and white, but whether with one or both eyes it was impossible to say, as the child was always alarmed at my presence, so that I was, to a great extent, forced to rely upon the investigations of the mother.

The improvement continued for a week or more after the operation, when the mother informed me that the child did not see so well as it had—that the change to her was very perceptible. Ten days after the first, a second operation was performed in the same way as the first, and was followed by a rapid improvement. The medication was continued as before. The eyes contined to improve, and a month after the last operation, as far as I could judge, were completely restored. Six months have now elapsed since the operation and the child has had no further difficulty.

If paracentesis corneæ renders iridectomy less hazardous as suggested by Prof. Sperino, and as the above operation and numerous others performed by other surgeons prove that it may result in permanent relief, why not in every favorable case commence the treatment with this operation?

